# Association of body mass index and smoking on outcome of Chinese patients with colorectal cancer

**DOI:** 10.1186/1477-7819-11-271

**Published:** 2013-10-12

**Authors:** Dan Liu, Qinggang Li, Zhenni Yang, Xiaocui Hu, Wenbiao Qian, Yaju Du, Bingrong Liu

**Affiliations:** 1 Department of Gastroenterology, Second Affiliated Hospital of Harbin Medical University, No. 246 Xuefu Road, Nangang, Harbin 150086, People’s Republic of China; 2 Department of Infectious Disease, First Affiliated Hospital of Harbin Medical University, Harbin 150086, People’s Republic of China

**Keywords:** Colorectal cancer, Mortality, Obesity, Smoking

## Abstract

**Background:**

The impact of body mass index (BMI) on the prognosis of patients with colorectal cancer remains largely unknown, particularly in Asian populations. Therefore, the aim of this study was to examine the influence of BMI on clinicopathological characteristics and mortality of Chinese colorectal cancer patients.

**Methods:**

The study cohort consisted of 525 patients who were diagnosed with colorectal cancer and underwent radical surgery at the second hospital of Harbin Medical University between June 2004 and August 2011. Study participants were divided into two BMI categories: normal weight (BMI <23 kg/m^2^) and overweight (BMI ≥23 kg/m^2^).

**Results:**

Of 525 patients, 208 patients (39.6%) were included in the normal-weight group and 317 patients were included in the overweight group. During the mean follow-up period of 48.8 months, 89 patients had disease recurrence and 131 deaths occurred. High BMI was significantly correlated with younger age, presence of diabetes, alcohol consumption, distal colon tumors, amount of lymph node harvested and pathological stage. No statistically significant correlation was found between high BMI and progression-free survival (PFS) or overall survival (OS) when the total group of patients was considered (*P* = 0.077 and *P* = 0.701, respectively). Cigarette-smoking patients had significantly shorter OS than patients who had never smoked (hazard ratio = 1.613, 95% confidence interval = 1.133 to 2.296; *P* = 0.008), and this difference in OS remained significant in multivariate analysis. Cigarette-smoking patients did not have significantly different PFS compared with patients who had never smoked.

**Conclusion:**

There was no significant correlation between obesity and outcomes of patients with colorectal cancer. In addition, our findings support the claims that cigarette smoking may be partially responsible for the divergent mortality of patients with colorectal cancer.

## Background

The prevalence of obesity and excessive weight has dramatically increased over previous decades, and a further increase is expected in the future [1]. A mounting body of epidemiological evidence indicates that westernized lifestyle and resulting overweight or obesity are responsible for the development of colorectal cancer [2-6]. The observation is possibly a result of increased insulin resistance and higher concentrations of insulin and insulin-like growth factor I, which promote cell proliferation and inhibit apoptosis [7-10]. Several molecular mechanisms have been proposed to explain the relation between body mass index (BMI) and colorectal cancer risk, including *CTNNB1* gene and tumor microsatellite instability status [11,12].

The impact of obesity on the prognosis of patients with colorectal cancer is complicated, and a limited number of studies indicate somewhat different effects [13-17]. Moreover, the association of BMI with morbidity of colorectal cancer has not been well-characterized in Asian populations [14,18]. To this end, we examined the association between BMI-defined obesity and the long-term survival of patients who underwent surgery for colorectal cancer in a Chinese population.

## Methods

### Patients

We retrospectively studied a total of 525 patients diagnosed with colorectal cancer who underwent radical surgery at the second hospital of Harbin Medical University between June 2004 and August 2011. Patients with any history of cancer and those who had familial adenomatous polyposis syndrome or hereditary nonpolyposis colorectal cancer were excluded from our study. Written consent was obtained from each patient, and the local ethics committee of second affiliated hospital of Harbin Medical University approved this study.

### Data collection

Data collection included family history, smoking history, history of alcohol consumption, history of diabetes mellitus, surgical outcomes, clinicopathological factors, chemotherapy administered and survival. Body weight was measured on the admission day. BMI was calculated as weight in kilograms divided by height in meters squared. According to the World Health Organization (WHO) classification for Asian populations, the patients were categorized as underweight (BMI <18.5), normal weight (18.5 ≤ BMI < 23.0), overweight (23.0 ≤ BMI < 27.5) or obese (BMI ≥27.5) [19]. Because the numbers of patients in the underweight group and the obese group were small, we divided all patients into two groups based on a BMI above or below 23.0. Patients were categorized according to whether they had ever or never smoked or had ever or never consumed alcohol.

Treatment of colon carcinoma was primarily by surgical resection with adjuvant chemotherapy for node-positive patients and node-negative patients with adverse pathological features. Most rectal cancer patients with T3 or T4 tumors were offered neoadjuvant therapy including a regimen of 5-fluorouracil and radiation therapy. After surgery, evaluation of fecal occult blood, carcinoembryonic antigen (CEA) measurement, chest X-ray, computed tomography (CT) and colonoscopy were carried out regularly. Recurrence was defined as the earlier date follow-up event, such as elevated CEA or abnormal findings on the CT scan or colonoscopy. The progression-free survival (PFS) and overall survival (OS) were defined from the date of diagnosis to the date of recurrence and death or the latest follow-up, respectively.

### Statistical analysis

Statistical analysis was performed using SPSS version 17.0 software (SPSS, Chicago, IL, USA). All *P* values are two-sided. Any difference in clinical features was assessed using a *χ*^2^ test, Mann–Whitney *U* test and analysis of variance for continuous variables. Survival was analyzed using the Kaplan-Meier method and compared using a logrank test.

## Results

### Baseline characteristics

Of 525 eligible patients, 28 patients (5.3%) were underweight (<18.5 kg/m^2^), 180 patients (34.3%) were normal weight (18.5 ≤ BMI < 23.0), 245 patients (46.7%) were overweight (23.0 ≤ BMI < 27.5) and 72 patients were obese (BMI ≥27.5). Two hundred eight patients (39.6%) were included in the normal-weight group (BMI <23.0), and 317 patients (60.4%) were included in the overweight group (BMI ≥23.0). Baseline characteristics and clinicopathological features are provided in Table 1.

**Table 1:** **Baseline characteristics of patients included in the study according to body mass index**^**a**^

**Characteristics**	**All cases**	**Normal weight**	**Overweight**	***P***
		**(BMI <23 kg/m**^**2**^**)**	**(BMI ≥23 kg/m**^**2**^**)**	
No. of patients (%)	525 (100%)	208 (39.6%)	317 (60.4%)	
Mean age (±SD), years	63.2 ± 11.7	64.5 ± 12.4	62.4 ± 11.1	0.003
Males, *n* (%)	310 (59%)	113 (36.5.0%)	197 (63.5%)	0.075
Diabetes, *n* (%)	86 (16.4%)	25 (12.0%)	61 (19.2%)	0.029
Alcohol consumption, *n* (%)	129 (24.6%)	41 (19.7%)	88 (27.8%)	0.036
Smoking, *n* (%)	160 (30.5%)	61 (29.3%)	99 (31.2%)	0.643
CEA ≥5 ng/ml, *n* (%)	215 (41.0%)	87 (41.8%)	128 (40.4%)	0.741
Site, *n* (%)				3.6 × 10^–4^
Rectum	287 (54.7%)	98 (47.1%)	189 (59.6%)	
Distal colon	116 (22.1%)	43 (20.7%)	73 (23.0%)	
Proximal colon	122 (23.2%)	67 (32.2%)	55 (17.4%)	
Tumor size, *n* (%)				0.513
<5 cm	332 (63.2%)	128 (62.5%)	204 (64.4%)	
≥5 cm	193 (36.8%)	80 (38.5%)	113 (35.6%)	
Pathological type, *n* (%)				0.166
Adenocarcinoma	462 (88.0%)	178 (85.6%)	284 (89.6%)	
others	63 (12.0%)	30 (14.4%)	33 (10.4%)	
Differentiation, *n* (%)				0.764
Well/moderate	436 (83.0%)	174 (83.7%)	262 (82.6%)	
Poor	89 (17.0%)	34 (16.3%)	55 (17.4%)	
Mean nodes analyzed (±SD)	13.6 ± 8.2	14.8 ± 9.0	12.9 ± 7.6	0.007
Lymph node-positive, *N* (%)	225 (42.9%)	79 (38.0%)	146 (46.1%)	0.067
Stage, *n* (%)				0.008
I	83 (15.8%)	26 (12.5%)	57 (18.0%)	
II	192 (36.6%)	90 (43.3%)	102 (32.2%)	
III	165 (31.4%)	53 (25.5%)	112 (35.3%)	
IV	85 (16.2%)	39 (18.8%)	46 (14.5%)	

^a^BMI, body mass index; CEA, carcinoembryonic antigen.

Higher BMI showed a significant correlation with younger age, presence of diabetes, alcohol consumption, distal site of colon tumor, less lymph node harvested and lower stage (Table 1). There were no significant differences between the two groups with regard to smoking, serum CEA, tumor size, pathological type and differentiation grade (Table 1).

### Body mass index, smoking and survival

**Figure 1:**
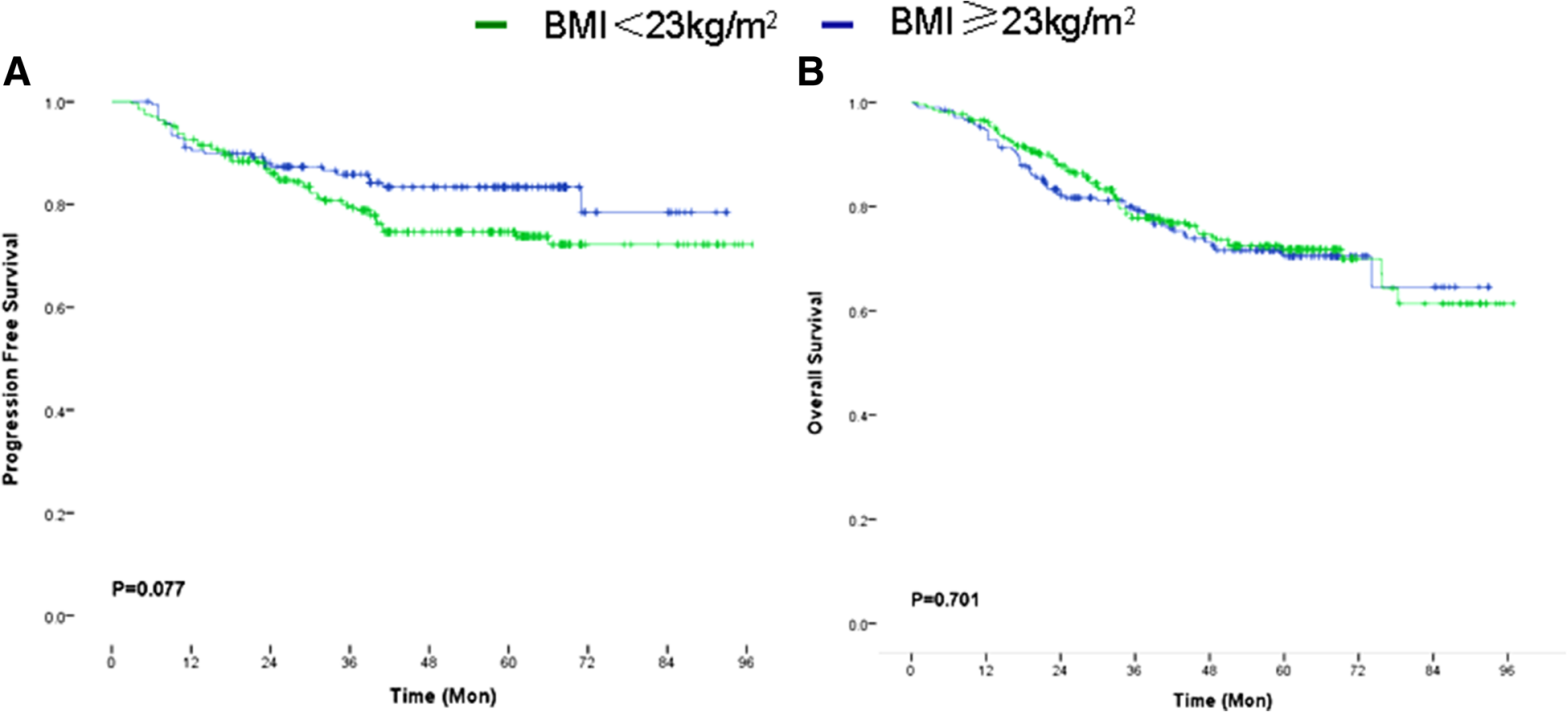
**Relationship between body mass index and clinical outcomes of colorectal cancer patients. A**, Kaplan-Meier survival curve demonstrating high BMI was not significantly related to progression-free survival; **B**, Kaplan-Meier survival curve demonstrating high BMI was not significantly related to overall survival.

In total, 89 patients (20.2%) without distant metastasis at diagnosis had disease recurrence after a median follow-up of 48.8 months (range = 5 to 97 months). The median PFS was 44 months (range = 3 to 97 months), and five-year OS was 75.0% for all stages in the whole series. During follow-up, a total of 131 patient deaths (25%) were encountered, with an even distribution between the BMI strata (26.0% vs. 24.3%; *P* = 0.665). No statistically significant correlation was found between high BMI and PFS or OS when the total group of patients was considered (*P* = 0.077 and *P* = 0.701, respectively) (Figure 1A and B, Table 2). The PFS and OS were also similar in the two BMI categories (*P* = 0.088 and *P* = 0.424, respectively), even in the 440-patient subgroup analysis, which included the patients without primary metastasis at the time of surgery. Even in the male and female subcohorts, PFS and OS were similar according to BMI categories (*P* = 0.476 and *P* = 0.407 for men, respectively; *P* = 0.110 and *P* = 0.933 for women, respectively).

**Table 2:** **Mean progression-free survival and overall survival for various characteristics based on Kaplan-Meier analysis**^**a**^

**Variables**	**Mean PFS**	***P***	**Mean OS**	***P***
	**(95% CI)**		**(95% CI)**	
BMI		0.077		0.701
<23 kg/m^2^	79.4 (74.6 to 84.2)		71.9 (66.9 to 76.9)	
≥23 kg/m^2^	77.2 (72.9 to 81.4)		75.0 (70.8 to 79.3)	
Mean age		0.628		0.727
<60 years	78.9 (74.0 to 83.8)		74.6 (69.6 to 79.6)	
≥60 years	78.3 (74.0 to 82.6)		74.0 (69.7 to 78.3)	
Gender		0.112		0.043
Male	75.3 (71.1 to 79.6)		71.1 (67.0 to 75.1)	
Female	81.3 (76.9 to 86.8)		77.3 (72.0 to 82.6)	
Diabetes		0.158		0.884
Yes	70.2 (61.9 to 78.5)		69.2 (60.5 to 77.8)	
No	80.3 (76.8 to 83.8)		74.9 (71.3 to 78.4)	
Alcohol		0.291		0.088
Yes	75.1 (68.3 to 81.9)		69.2 (62.6 to 75.8)	
No	80.1 (76.4 to 83.7)		76.2 (72.4 to 80.0)	
Smoking		0.222		0.007
Yes	74.7 (68.5 to 80.9)		67.9 (62.0 to 73.8)	
No	80.3 (76.5 to 84.1)		76.9 (72.9 to 80.8)	
Site		0.289		0.788
Colon	78.4 (74.0 to 82.8)		73.2 (68.8 to 77.7)	
Rectum	77.5 (73.0 to 82.1)		74.0 (69.5 to 78.5)	
Tumor size		0.903		0.015
<5 cm	79.8 (75.8 to 83.8)		78.1 (74.0 to 82.3)	
≥5 cm	77.4 (72.0 to 82.9)		69.2 (64.0 to 74.4)	
Pathological type		0.291		2.5 × 10^–4^
Adenocarcinoma	79.9 (76.5 to 83.3)		76.7 (73.2 to 80.3)	
Others	71.7 (61.5 to 82.0)		59.2 (50.2 to 68.3)	
Differentiation		0.112		4.0 × 10^–5^
Grade 3/4	80.4 (77.0 to 83.9)		78.0 (74.6 to 81.4)	
Grade 1/2	67.3 (59.1 to 75.4)		57.1 (49.7 to 64.5)	
Lymph nodes harvested		0.910		0.575
≥12	79.3 (74.4 to 84.2)		73.4 (68.4 to 78.5)	
<12	76.3 (72.2 to 80.3)		73.0 (69.0 to 77.1)	
Lymph nodes positive		1.5 × 10^–7^		2.0 × 10^–13^
Positive	67.8 (61.9 to 73.8)		60.9 (55.6 to 66.3)	
Negative	85.4 (81.8 to 89.0)		84.6 (80.9 to 88.3)	

^a^BMI, body mass index; CI, confidence interval; OS, overall survival; PFS, progression-free survival.

**Figure 2:**
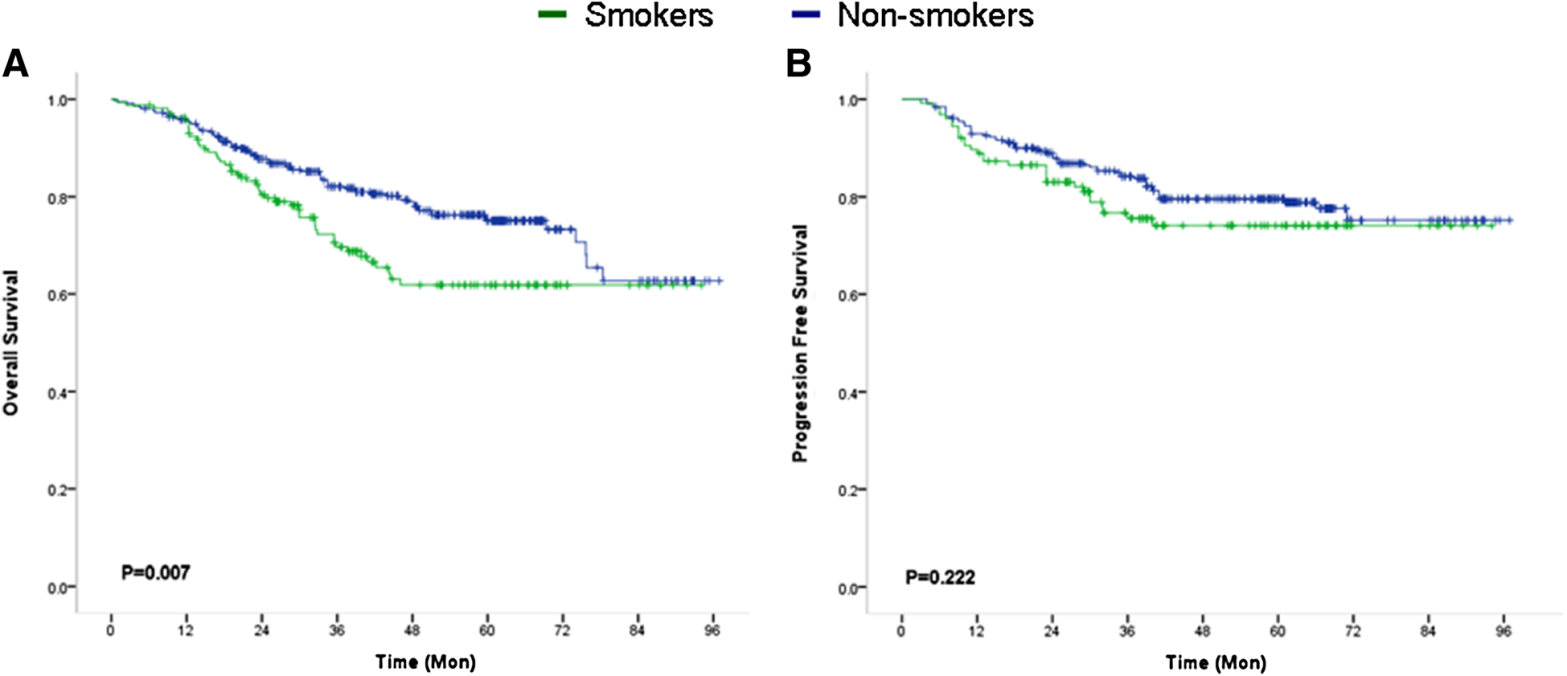
**Relationship between smoking and clinical outcomes of colorectal cancer patients. A**, Kaplan-Meier survival curve demonstrating cigarette-smoking was related to shorter overall survival; **B**, Kaplan-Meier survival curve demonstrating cigarette-smoking was not related to progress-free survival.

The BMI and cigarette smoking interaction was not significant (*P* = 0.322), indicating that smoking status was not significantly related to BMI. In both sexes, univariate analysis revealed that cigarette-smoking patients had significantly shorter OS than patients who had never smoked (hazard ratio = 1.613, 95% confidence interval = 1.133 to 2.296; *P* = 0.007) (Figure 2A, Table 2). Cigarette-smoking patients did not have significantly different PFS from patients who had never smoked (*P* = 0.222) (Figure 2B, Table 2). In this regard, only 20.8% of patients who had never smoked died as a result of colorectal cancer five years after surgery, as compared with 31.3% of patients who had smoked. In multivariable analysis, smoking status was a potentially independent factor in OS (*P* = 0.026) after adjusting for gender, tumor size, pathological type and differentiation.

## Discussion

The prevalence of excessive weight and obesity worldwide has been increasing markedly over the past decades [20]. Although the prevalence of obesity remains low in many Asian countries compared with Western countries, many Asian countries are experiencing a dramatic rise in the incidence of obesity [1]. Excessive weight and obesity are associated with higher risk of many cancers, including colorectal cancer; however, few studies have focused on the association between obesity and outcomes of cancer patients. Moreover, in Asian populations, only two published studies have examined the effect of obesity on prognosis in patients with colorectal cancer [14,18].

The results of the limited number of studies regarding the influence of BMI on the outcomes of patients with colorectal cancer are often unclear, even if conducted in the same population. For instance, in a study that comprised a total of 509 Korean colorectal cancer patients, excessive weight had a favorable influence on OS compared with the results obtained for normal-weight participants (*P* = 0.001), whereas there was no significant difference in PFS (*P* = 0.735) [14]. Contrary to this finding, Moon *et al*. reported that there was no association between BMI and OS (*P* = 0.210), but the overweight group showed a borderline decrease in disease-free survival compared with normal-weight participants (*P* = 0.064) [18]. These discrepancies may result from the difference of BMI category between the two study populations, aside from the disparity in sample sizes. In the first study, the participants were divided into normal-weight and overweight groups based on BMI of 23 kg/m^2^, which is the criterion set for Asian populations. In the latter study, the participants were divided based on a BMI of 25 kg/m^2^ according to WHO criteria for excessive weight. In our study, we divided 525 Chinese participants based on BMI above or below 23 kg/m^2^ according to the WHO classification for Asian populations. BMI had no influence on OS or PFS of patients who underwent surgery for colorectal cancer, which is inconsistent with the two previous studies.

The influence of BMI on the prognosis of patients with colorectal cancer is not clear, with the few studies that have addressed this question showing somewhat disparate findings. Dignam *et al*. observed that BMI greater than 35 kg/m^2^ at diagnosis was associated with an increased risk for recurrence and death from colon cancer [21]. In the study by Sinicrope *et al*., obesity was further categorized as class 1 (BMI = 30 to 34.9 kg/m^2^) and class 2/3 (BMI ≥35 kg/m^2^) [16]. In the class 2/3 obesity group, patients had worse PFS and OS rates compared with normal-weight patients. Moreover, patients in this group showed a trend toward worse PFS in multivariate analysis. One possible explanation for the difference between Western studies and ours relates to the inclusion of more obese participants in Western cohorts. Nevertheless, the Irish study showed no significant difference between obese and nonobese cohorts regarding survival, which is consistent with our results.

In our study, we found that both past and current smokers had higher rates of mortality (OS) than patients who had never smoked. In multivariate analysis, patients who had smoked had a 10% increase in the risk of death after surgery compared with the nonsmoking cohort. In contrast to our study, the US population-based Cancer Prevention Study II found that men and women who smoked cigarettes for 20 or more years experienced higher colorectal cancer death rates, adjusted for multiple potential confounders [22]. However, Colangelo *et al*. reported that the association between cigarette smoking and colorectal cancer mortality was stronger in the younger age group (age <50 years) than in the older age group [23]. In this study, we did not further stratify the patients by smoking status, because it was recorded only at baseline.

## Conclusion

Our data fail to suggest any significant impact of obesity on outcomes of colorectal cancer patients. These findings support the proposal that cigarette smoking may be partially responsible for divergent colorectal cancer mortality. Besides, there are some limitations that should be considered. These analyses were based on a retrospective study design, so the potential for selection bias and missing data, including medical history and comorbidities, may have affected data analysis. Furthermore, BMI is the sole marker of obesity and nutritional status. Baseline information on anthropometric measures of body weight was not available. To better understand the impact of obesity and smoking on the outcome of colorectal cancer patients, more large-scale prospective studies are necessary.

## Competing interests

The authors declare that they have no competing interests.

## Authors’ contributions

DL and QL carried out statistical analysis and drafted the manuscript. ZY, XH, WQ and YD participated in the data collection and follow up of all the patients. BL designed this study and revised the initial manuscript. All authors read and approved the final manuscript.
